# Fellow-Workers and the Roll of Honour

**Published:** 1915-02-27

**Authors:** 


### ROUND THE WORLD.

[The Editor invites Early Information and Contributions Marked "Fellow-Workers."]

### Fellow-Workers and the Roll of Honour.

The department for diseases of the throat, nose, and ear at the King George Hospital is in charge of the four following specialists—namely, Mr. A. H. Cheatle, Mr. Dundas Grant, Sir St. Clair Thomson, and Mr. Herbert Tilley.

Mr. Arthur Henry Cheatle, F.R.C.S. Eng., is aural surgeon at King's College Hospital, and lecturer on aural surgery at the medical school attached thereto. One of our leading specialists on diseases of the ear, he is a Past-President of the otological section of the Royal Society of Medicine, and occupied the chair in the section of ear diseases at the last International Medical Congress. His interest in the problem of gun deafness and its prevention—a subject on which he has written—should serve him in good stead in his new work. Mr. Cheatle, who was at one time aural surgeon to King Edward VII.'s Hospital, and Hunterian professor at the Royal College of Surgeons, won the Adam Politzer prize at the ninth International Otological Congress.

Mr. J. Dundas Grant, M.D. Edin., F.R.C.S. Eng. and Edin., is throat and ear surgeon to the Brompton Hospital for Consumption and the West End Hospital for Nervous Diseases, as well as consulting surgeon to the Central London Throat and Ear Hospital, which he has served actively for many years. A keen musician, he is also surgeon to the Royal Academy of Music, and aural surgeon to the Royal Society of Musicians, and has for a long time found his chief hobby in orchestral music. After studying medicine at Edinburgh and "Bart.'s," Mr. Dundas Grant spent ten years in general practice before establishing himself as a specialist.

Sir St. Clair Thomson, who was knighted three years ago, is professor of laryngology, and physician for diseases of the throat, at King's College Hospital, and a Vice-President of the Medical Society of London. A student of King's College Hospital, he obtained the academic distinctions of M.D. Lond., F.R.C.P. Lond., and F.R.C.S. Eng., in addition to which he is an M.D. of Lausanne. It will be remembered that Sir St. Clair Thomson was called in during the last illness of the late King. At one time he was physician to the Throat Hospital in Golden Square, surgeon to the throat and ear department at the Seamen's (*Dreadnought*) Hospital, surgeon to the Royal Ear Hospital, and professor of laryngology and otology at the Royal Army Medical College.

Mr. Herbert Tilley, M.D. Lond., F.R.C.S. Eng., entered University College Hospital as a student, and distinguishing himself there became ultimately a member of the staff, and is now surgeon to the ear and throat department of that institution. At one time he was surgeon to the Throat Hospital in Golden Square. Mr. Tilley lectures on diseases of the ear, nose, and throat at the Royal Army Medical College, and is also laryngologist to the Radium Institute. The author of a well-known text-book on "Diseases of the Throat and Nose," he is now president of the laryngological section of the Royal Society of Medicine.

Cornwall has lost a capable sanitarian by the death of Lieutenant-Colonel Robert Burnet, R.A.M.C. (T.), as a result of an accident. Originally a student of University College, Liverpool, he possessed himself of a lengthy string of diplomas, including the M.Sc., M.B., Ch.B. Vict., M.Sc., M.B., Ch.B. Liverpool, and the D.P.H. Birm. Soon after taking his public health diploma in 1908 Lieutenant-Colonel Burnet was elected medical officer of health for Bury, Lancashire, and subsequently was made superintendent of the Infectious Diseases Hospital there. About the same time he became interested in the Territorial branch of the R.A.M.C., and obtained a commission therein, and gave such keen attention to his military duties that he received rapid promotion. Five years ago he was appointed medical officer of health for Cornwall, and almost immediately had to combat the outbreak of polio-myelitis which, it may be remembered, occurred at that time.

Mr. Hugh Lett, M.B. Vict., F.R.C.S. Eng., who has been doing important surgical work at the Anglo-American Hospital for wounded at Wimereux, is a Leeds man who qualified from the Victoria University, and, coming up to town, worked at the London Hospital, where he has been surgical tutor and demonstrator in anatomy, and is now full surgeon and joint lecturer on operative surgery. His present appointments also include those of honorary consulting surgeon to the Bushey Heath Cottage Hospital and to the Ilford Emergency Hospital, whilst before his election to the staff of the "London" he was assistant surgeon to the Belgrave Hospital for Children. Some little time ago Mr. Lett published an interesting analysis of a thousand cases of appendicitis.

Lieutenant-Colonel Michael Joseph Whitty, who retired some little time ago, takes up active service again with his new appointment as medical officer for recruits in the Western Command. Lieutenant-Colonel Whitty was a student of Queen's College, Cork, and the Catholic University in Dublin; obtaining the licence in midwifery of the Coombe Hospital in 1884, he became an M.D., M.Ch. R.U.I., in the following year.

The Red Cross Hospital at Worthing is medically staffed by four well-known local practitioners—namely, Dr. J. R. Lunn, Dr. H. Wiggins, Dr. R. Smedley, and Dr. Brown. Mr. John R. Lunn, F.R.C.S. Eng. and Edin., has been in practice at Worthing for many years, and was formerly house surgeon to St. Thomas's Hospital and assistant medical officer to the East London Hospital for Children. Subsequently he was for some time resident medical superintendent to the St. Marylebone Infirmary. Mr. Henry Wiggins, M.R.C.S. Eng., L.R.C.P. Lond., studied medicine at Charing Cross Hospital, where he was electrical assistant before he settled down in general practice. Whilst Dr. Ralph Smedley, M.D. Cantab., M.R.C.S. Eng., L.R.C.P. Lond., D.P.H., is a Cambridge and "Guy's" man, who now holds the appointments of medical officer for health and school medical officer for West Sussex.

